# First Whole-Genome Sequence of a Highly Resistant Klebsiella pneumoniae Sequence Type 14 Strain Isolated from Sudan

**DOI:** 10.1128/MRA.00552-19

**Published:** 2019-08-08

**Authors:** Sofia B. Mohamed, Sumaya Kambal, Abdalla Munir, Nusiba Abdalla, Mohamed Hassan, Ahmed Hamad, Sara Mohammed, Fatima Ahmed, Omnia Hamid, Arshad Ismail, Mushal Allam

**Affiliations:** a Bioinformatics and Biostatistics Department, National University Biomedical Research Institute, National University, Khartoum, Sudan; b Sequencing Core Facility, National Institute for Communicable Diseases, National Health Laboratory Service, Johannesburg, South Africa; Georgia Institute of Technology

## Abstract

Klebsiella pneumoniae is an opportunistic pathogen that accounts for a significant proportion of hospital-acquired infections and is a leading cause of nosocomial outbreaks. Here, we describe the genomic sequence of a highly resistant K. pneumoniae sequence type 14 (ST14) strain isolated from Sudan.

## ANNOUNCEMENT

Klebsiella pneumoniae is a Gram-negative bacterium well known as an opportunistic pathogen that causes pneumonia, septicemia, and urinary tract infection (1). Nosocomial outbreaks of multidrug-resistant *Klebsiella* spp. are often caused by extended-spectrum β-lactamase (ESBL) producers. The emergence of ESBL-producing strains among clinical *Klebsiella* isolates has been progressively increasing over the years with very limited therapeutic options (2). Here, we report the draft genome sequence of a highly resistant ESBL K. pneumoniae strain (NUBRI-K) isolated from Sudan.

A sample of sputum was collected from a 44-year-old female who was admitted to the Omdurman Teaching Hospital in Khartoum, Sudan, with a pneumonia infection. The specimen was directly inoculated onto MacConkey agar and, afterward, incubated overnight under aerobic conditions at 37°C. The colony was identified using Gram staining and biochemical tests that included oxidase, catalase, kligler’s iron agar (KIA), sulfide indole motility, citrate agar, and urea tests. The analytical profile index was used to confirm the species (3). Disk diffusion testing was carried out on the isolate according to the Clinical and Laboratory Standards Institute (CLSI; M100 2007) guidelines (4). The genomic DNA was extracted using a QIAamp DNA minikit (Qiagen, Germany). Paired-end libraries were prepared using the Nextera DNA flex library prep kit, followed by 2 × 300-bp sequencing on the MiSeq platform (Illumina, Inc., USA). The resultant paired-end reads were quality trimmed using Sickle version 1.33 (with the parameters -q 20 and -l 75) (5) and *de novo* assembled using SPAdes version 3.11 (with the parameters careful and cutoff auto) (6). The contiguous sequences were then submitted to the NCBI Prokaryotic Genome Annotation Pipeline (7). The multilocus sequence types (MLST) (8), resistance genes, and plasmids were predicted using ResFinder (9) and PlasmidFinder (10) through the GoSeqIt tools Web platform (9). Virulence factors were determined using the VirulenceFinder database (11). Default settings were used in all software unless otherwise noted.

A total of 1,493,916 paired-end reads were obtained from the whole-genome sequence of strain NUBRI-K. Quality-controlled reads (1,486,286 reads, average length of 198.6 bp) with a Phred score of >20 were assembled *de novo* with a minimum contig cutoff of 200 bp to 137 contigs (smallest contig, 223 bp; largest contig, 490,613 bp; *N*_50_, 267,178 bp; and 52× genome coverage). The genome total length was 5,880,496 bp, with a G+C content of 56.6%. In total, the NUBRI-K genome contains 5,988 genes, including 5,895 protein-coding genes and 93 RNA genes. The MLST was deﬁned as sequence type 14 (ST14). A total of 24 acquired antibiotic resistance genes were found in NUBRI-K, including aminoglycoside resistance genes [*aac(3)-Iia*, *aac(6′)-Ib-cr*, *aph(3″)-Ib*, *aph(3′)-Ia*, *aph(6)-Id*, and *armA*], β-lactam resistance genes (*bla*_CTX-M-15_, *bla*_OXA-1_, *bla*_SHV-28_, and *bla*_TEM-1B_), fluoroquinolone resistance genes [*aac (6′)-Ib-cr*, *oqxA*, *oqxB*, and *qnrB1*], a fosfomycin resistance gene (*fosA*), macrolide resistance genes *mdf*(A), *mph*(A), *mph*(E), and *msr*(E), a phenicol resistance gene (*catB3*), sulphonamide resistance genes (*sul1* and *sul2*), and trimethoprim resistance genes (*dfrA14* and *dfrA5*) which showed between 99.77% and 100% identity to query sequences in the ResFinder database. The NUBRI-K genome harbored a total of six plasmids [Col440I, ColpVC, IncFIB(K), IncFIB(Mar), IncFII, and IncHI1B] which showed between 89.47% and 99.54% identity to query sequences in the PlasmidFinder database. Col440I and ColpVC are circular plasmids. In the NUBRI-K genome, 131 virulence determinants were identified, namely, 19 adherence and biofilm-formation genes, 1 antiphagocytosis gene, 2 efflux pump genes, 34 iron acquisition genes, 6 nutritional factor genes, 4 regulation genes, 46 secretion system genes, 1 serum resistance gene, and 18 toxin genes.

### Data availability.

The draft whole-genome project for NUBRI-K has been deposited at DDBJ/EMBL/GenBank under accession number SOYT00000000. Raw sequence reads have been deposited at DDBJ/EMBL/GenBank under BioProject accession number PRJNA526408.

## References

[1] Bishara J, Leibovici L, Huminer D, Drucker M, Samra Z, Konisberger H, Pitlik S 1997 Five-year prospective study of bacteraemic urinary tract infection in a single institution. Eur J Clin Microbiol Infect Dis 16:563–567. doi:10.1007/BF02447917. 9323466

[2] Livermoore DM, Paterson DL 2006 Pocket guide to extended spectrum β-lactamases in resistance. Springer (India) Private Limited, New Delhi, India.

[3] Betty AF, David F, Alice SW 2007 Diagnostic microbiology, 12th ed Andrew Allen, Chicago, IL.

[4] Ferao MJ, Craig WC, Dudlley MN 2002 Performance standards for antimicrobial susceptibility test, p 15–17. CLSI, Wayne, PA.

[5] Joshi NA, Fass JN 2011 Sickle: a sliding-window, adaptive, quality-based trimming tool for FastQ files (version 1.33). https://github.com/najoshi/sickle.

[6] Bankevich A, Nurk S, Antipov D, Gurevich AA, Dvorkin M, Kulikov AS, Lesin VM, Nikolenko SI, Pham S, Prjibelski AD, Pyshkin AV, Sirotkin AV, Vyahhi N, Tesler G, Alekseyev MA, Pevzner PA 2012 SPAdes: a new genome assembly algorithm and its applications to single-cell sequencing. J Comput Biol 19:455–477. doi:10.1089/cmb.2012.0021. 22506599 PMC3342519

[7] Tatusova T, DiCuccio M, Badretdin A, Chetvernin V, Nawrocki EP, Zaslavsky L, Lomsadze A, Pruitt KD, Borodovsky M, Ostell J 2016 NCBI Prokaryotic Genome Annotation Pipeline. Nucleic Acids Res 44:6614–6624. doi:10.1093/nar/gkw569. 27342282 PMC5001611

[8] Larsen MV, Cosentino S, Rasmussen S, Friis C, Hasman H, Marvig RL, Jelsbak L, Sicheritz-Pontén T, Ussery DW, Aarestrup FM, Lund O 2012 Multilocus sequence typing of total-genome-sequenced bacteria. J Clin Microbiol 50:1355–1361. doi:10.1128/JCM.06094-11. 22238442 PMC3318499

[9] Zankari E, Hasman H, Cosentino S, Vestergaard M, Rasmussen S, Lund O, Aarestrup FM, Larsen MV 2012 Identification of acquired antimicrobial resistance genes. J Antimicrob Chemother 67:2640–2644. doi:10.1093/jac/dks261. 22782487 PMC3468078

[10] Carattoli A, Zankari E, García-Fernández A, Voldby Larsen M, Lund O, Villa L, Møller Aarestrup F, Hasman H 2014 *In silico* detection and typing of plasmids using PlasmidFinder and plasmid multilocus sequence typing. Antimicrob Agents Chemother 58:3895–3903. doi:10.1128/AAC.02412-14. 24777092 PMC4068535

[11] Lihong C, Zheng DD, Liu B, Yang J, Jin Q 2016 VFDB: hierarchical and refined dataset for big data analysis—10 years on. Nucleic Acids Res 44:D694–D697. doi:10.1093/nar/gkv1239. 26578559 PMC4702877

